# Intracardiac Thrombosis: An Uncommon Manifestation of Behçet’s Disease

**DOI:** 10.7759/cureus.44385

**Published:** 2023-08-30

**Authors:** Rhita Ezzahraoui, Najlaa Belharty, Zaineb Bourouhou, Hajar EL Ouartassi

**Affiliations:** 1 Cardiology, Mohammed V University, Rabat, MAR; 2 Cardiology B, Ibn Sina University Hospital Center, Rabat, MAR; 3 Cardiology B, Mohammed V University, Rabat, MAR

**Keywords:** anticoagulation, case report, echocardiography, cardiac thrombus, behcet

## Abstract

Behçet’s disease is a chronic multisystemic vasculitis of unknown etiology that evolves in relapses and remissions with a predominance of cutaneous and ocular lesions. It is more frequent in young adults, with a predominantly male population from the Mediterranean and Middle East.

Cardiac involvement is rare (1 to 6%) but represents one of the most serious complications that can affect the three tunics of the heart. Cardiac thrombus is uncommon, its diagnosis relies mainly on echocardiography, and the development of the empirical treatment is based on immunosuppressive therapy and anticoagulation depending on the case.

We report the case of a 21-year-old man admitted for a 2-month history of fever, loss of weight, and body condition in association with an intracardiac thrombus in the context of Behçet's disease.

Intracardiac thrombus is a rare manifestation of Behçet's disease; the diagnosis must be made quickly in order to allow early management.

## Introduction

Behçet's syndrome is a systemic disease characterized by bipolar aphthosis and ophthalmic involvement and can also affect several other sites (cutaneous, pulmonary, gastrointestinal, genitourinary, central nervous system, and cardiovascular) which could influence the prognosis of the disease [1]. Cardiac involvement is rare, with serious consequences: intracardiac thrombosis, cardiomyopathy, pericarditis, valvulopathy, acute coronary syndrome, and conduction disorders [2]. Despite its sporadic occurrence, it is strongly correlated with mortality.

We report the case of a young man admitted for management of fever with loss of body weight, a condition that was present for two months.

## Case presentation

Patient information

A 21-year-old Moroccan man with no past medical history sought medical attention for a 2-month history of fever of unknown origin and weight loss. he experienced severe exertion that limited his mobility. Medical history revealed previous attacks of intermittent shivers together with recurrent genital ulcers.

Clinical findings

The initial physical examination revealed a temperature of 38°C and multiple scrotal ulcerations.

Diagnostic assessment

The laboratory tests indicated anemia, hemoglobin at 8.3 g/dL, neutrophil leukocytosis at 12250/L, and C-reactive protein at 246 mg/dL. Haemocultures were negative, as was the bronchial aspirate culture for mycobacteria.

Body computed tomography (CT) has been conducted and revealed no anomaly, except for chest ground glass opacities suggesting an infectious origin. There was no evidence of pulmonary embolism, vasculitis, and pulmonary aneurysm in CT pulmonary angiography.

The patient received wide-spectrum antibiotics, yet, showed no clinical improvement.

**Figure 1:**
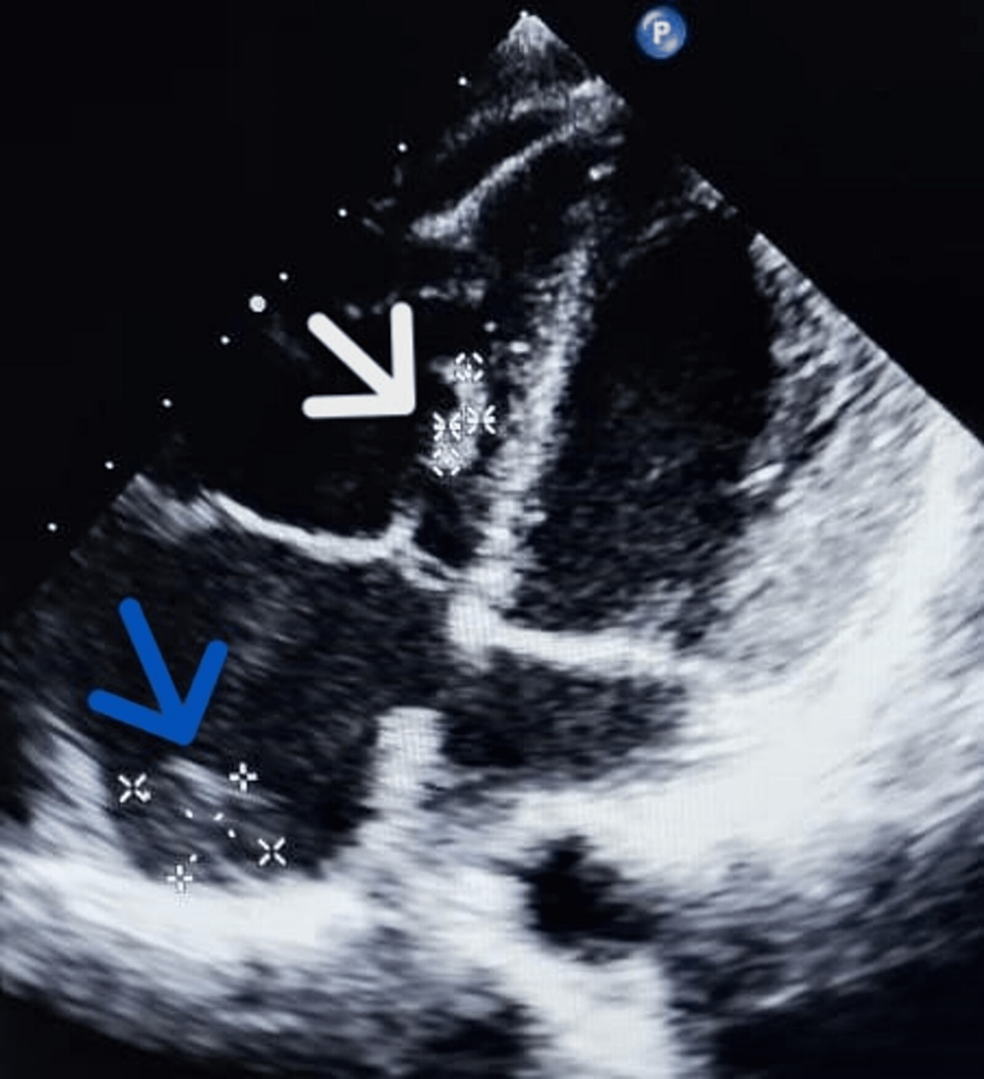
Transthoracic echocardiography, in apical four-chamber view, demonstrating a right ventricular thrombus 7×15 mm (white arrow), and a large right atrium thrombus 14×18 mm (blue arrow)

**Figure 2:**
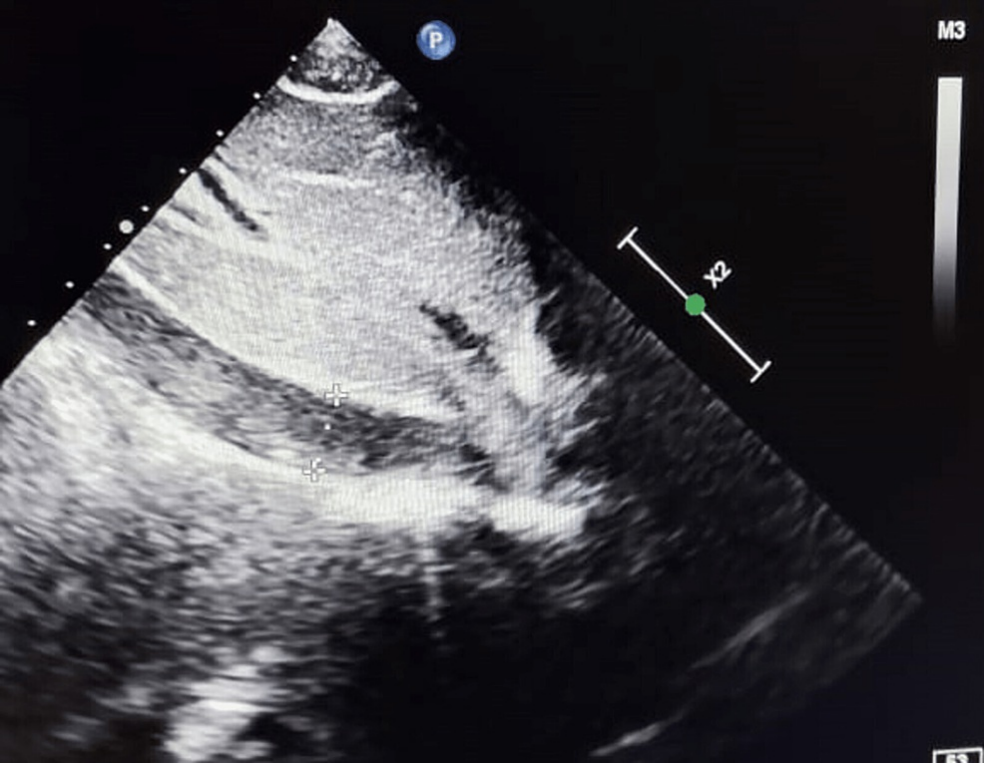
In the subcostal view of TTE, the inferior vena cava appears filled with thrombus TTE: transthoracic echocardiography

We performed transthoracic echocardiography (TTE) that detected a 7×15 mm hyperechogenic mass located in the right ventricle, adjacent to the interventricular septum, and a right atrial echogenic mass of 14×18 mm consistent with thrombus (Figure 1). Besides, TTE exploring the inferior vena cava (IVC), revealed an intraluminal mass consistent with thrombus causing turbulence of flow on color Doppler (Figure 2).

He had no family history of coagulopathy and the coagulation tests (prothrombin time, activated partial thromboplastin time, and bleeding time) and protein C, protein S, and antithrombin III levels were within normal limits. Antinuclear antibody (ANA), anticardiolipin antibody, lupus anticoagulant, and rheumatoid factor (RF) were also negative.

With the exclusion of infection, it was assumed that the intracardiac masses and the IVC thrombus were secondary to heart and venous involvement by Behçet's disease respectively, taking into consideration the patient’s history of recurrent genital ulcers, the presence of the scrotal ulceration scars, and a pathergy test that came out positive.

Therapeutic intervention and follow-up

The patient received intravenous high-dose methylprednisolone followed by oral prednisone and anticoagulation therapy.

After 2 months, the intracardiac thrombi substantially decreased in size on echocardiography along with the gradual resolution of the mass in IVC.

## Discussion

In Behçet's disease, cardiac localization is rare, in the order of 1 to 6%. Cardiovascular complications may have fatal consequences and sometimes constitute the first mode of revelation of the disease with a poorer prognosis compared to lesions in other organs.

The three tunics of the heart can be affected: pericardial involvement is the most frequent (20% to 40%), then the myocardial involvement (20%), often manifested by myopericarditis, and finally, the endocardial involvement can be confined to the valves, or extend to the ventricular wall [3]. Endomyocardial fibrosis may be discovered incidentally on echocardiography or revealed by a right heart decompensation, more often affecting the right heart with a thickened endocardium.

Cardiac involvement may also concern the coronary arteries and be responsible for coronary syndrome in young subjects [4,5].

In our case, the cardiac involvement was manifested by an intracavitary thrombus. This rarely inaugural involvement is generally isolated or associated with pulmonary artery aneurysms, venous thrombosis, or endomyocardial fibrosis. It can involve both the atria and the ventricles, with the right ventricle as the predilection site, which can lead to pulmonary embolism [1].

The pathophysiology of thrombotic predisposition is still unknown. Several mechanisms have been suggested:

Ischemia or endothelial cell rupture, the presence of antiphospholipid antibodies, prothrombotic plasma factors, and deposition of immune complexes in the blood vessel [6].

Although vascular involvement is not uncommon in Behçet's disease (about one-third of patients), cardiac involvement, and in particular intracavitary thrombus, remains an exceptional manifestation of the disease [7].

The diagnosis is mainly evoked by transthoracic and transesophageal echography, which reveal the thrombus as an intracavitary mass, most often of ventricular location, heterogeneous, adherent to the wall. The differential diagnosis is myxoma or endomyocardial fibrosis [5].

MRI can distinguish between a tumor and an intracardiac thrombus, and in the case of endomyocardial fibrosis, it represents the gold standard for diagnosis showing a 3-layer appearance: a healthy outer myocardial layer, an intermediate layer of fibrosis, and finally internal thrombotic structures [8].

The clinical context and regression under anticoagulant therapy are diagnostic arguments.

Regarding the treatment of cardiac thrombosis in Behçet's syndrome, there is no consensus to date on the best approach to follow. Evidence for treatment is based on case series reported in the literature, the majority of which were treated with a combination of anticoagulants and immunosuppressants (azathioprine or cyclophosphamide) with superior results [2]. Anticoagulants should be used with caution or avoided in cases of pulmonary artery aneurysms because of the risk of hemoptysis. In the presence of extensive or recurrent thrombosis despite medical treatment or cardiac congestion, cardiac surgery may be considered [1].

## Conclusions

Cardiac involvement in Behçet's disease is an infrequent and underestimated manifestation that conditions the prognosis. The discovery of an intracardiac mass in a young subject should lead to the diagnosis of intracardiac thrombus in the context of Behçet's disease. Echocardiography is essential, and treatment is based mainly on immunosuppressants and anticoagulation.
